# Early maternal obesity shapes offspring development through immune mechanisms: a systematized review

**DOI:** 10.3389/fimmu.2026.1730730

**Published:** 2026-06-03

**Authors:** Jiale Zhang, Yu Chen, Zhijie Hu, Chaoqun Ding, Jing Chen, Yuxin Chen

**Affiliations:** 1 Department of Laboratory Medicine, Joint Institute of Nanjing Drum Tower Hospital for Life and Health, College of Life Science, Nanjing Normal University, Nanjing, Jiangsu, China; 2 Department of Laboratory Medicine, Nanjing Drum Tower Hospital Clinical College of Nanjing Medical University, Nanjing, Jiangsu, China; 3 Department of Laboratory Medicine, Nanjing Drum Tower Hospital, Nanjing University Medical School, Nanjing, Jiangsu, China; 4 Department of Laboratory Medicine, Nanjing Drum Tower Hospital Clinical College of Nanjing University of Chinese Medicine, Nanjing, Jiangsu, China

**Keywords:** cytokines, immune, inflammation, maternal obesity, offspring, pre- pregnancy

## Abstract

In recent years, rates of maternal obesity before conception have continued to rise globally. Maternal obesity in early pregnancy may alter intrauterine immune mechanisms, impact fetal metabolic programming, and increase the risk of later-life obesity. This study aimed to review the literature on changes in immune parameters and weight-related outcomes in offspring reported in human and animal studies of maternal obesity. A systematized literature search was conducted using PubMed and Web of Science for relevant human and animal studies published between 2010 and 2025. Study screening was facilitated using Rayyan. The risk of bias was assessed using SYRCLE for animal studies and LEGEND for human studies. Fifteen studies reporting birth weight outcomes showed conflicting findings, with three reporting higher birth weight, three reporting lower birth weight in offspring of mothers with versus without obesity, eight reporting no significant association, and one study did not report a p-value. These discrepancies may reflect heterogeneity in study models (e.g., *Sprague–Dawley* rats*, C57BL/6J* mice, and rhesus macaques), dietary compositions (e.g., cafeteria diet, Western-style diet, and high-fat diet), and species (animal versus human studies). In animal studies, gene expression analyses across different species and sample sources consistently showed upregulation of TLR2, ARG1, VEGF, TNF, and NF-κB, but variable trends for TNF-α, IL-10, IL-6, TLR4, TRAF6, STAT3, and MCP-1(CCL2). In human studies, placental gene expression of IL-1β, MCP-1 (CCL2), CXCR2, STAT3, P38-MAPK, and IL-8 varied across studies, while blood levels of IL-6, CRP, microRNAs, and antibodies also showed inconsistency. Placental macrophage and neutrophil counts were increased in humans, whereas animal studies consistently reported increased placental macrophage counts. Maternal pre-pregnancy or first trimester obesity showed inconsistent effects on offspring birth weight; however, it is associated with alterations in multiple immune parameters and may induce placental inflammation via the macrophage TLR2–NF-κB–p38 signaling pathway.

## 1 Introduction

Malnutrition is broadly defined as an imbalance between the body’s nutrient or energy requirements and intake (1). It encompasses three major groups: undernutrition and micronutrient-related malnutrition, and overnutrition. Traditionally, malnutrition was primarily equated with undernutrition, typically presenting as stunting (low-height-age), wasting (low weight-for-height), or underweight (low-weight-for-age). Global economic growth and changing dietary and lifestyle patterns, particularly high-calorie food intake and sedentary behaviors, have contributed to the emergence of a new malnutrition paradigm: overweight (high weight-for-height) and obesity. Obesity is associated with an increased risk of diet-related noncommunicable diseases (NCDs) such as cardiovascular disease and metabolic disease, which are rapidly becoming a dominant public health concern worldwide (1, 2). Obesity denotes pathologically elevated body fat that can harm health (3). Body mass index (BMI), defined as weight in kilograms over height in meters squared, is the routine metric for weight categorization (4). Adults with BMI 25.0–29.9 kg/m² are overweight, ≥ 30.0 kg/m² obese, and ≥ 40.0 kg/m² severely obese (5). More than one billion people worldwide are living with obesity (6). In 2019, an estimated 5 million deaths worldwide were attributable to high BMI-related NCDs (3). By 2030, the United Kingdom is expected to have the highest obesity prevalence in Europe, with obesity rates in adults forecast to surpass 35% (7). The increasing prevalence of obesity is particularly notable among women. In 2024, 26.2% of pregnant women in England were classified as obese during early pregnancy (8). Maternal pre-pregnancy or first trimester obesity (MO) may be related to increased risks of neonatal macrosomia (a birth weight of ≥ 4,000 grams), low birth weight (a birth weight of < 2500 grams), fetal growth restriction, as well as maternal complications such as preeclampsia, gestational diabetes, labor induction, Intensive Care Unit admission, and reduced spontaneous vaginal delivery rates (9–12). Evidence from multiple studies suggests that both low birth weight (<2,500 grams) and high birth weight (>3,500–4,000 grams) are associated with an elevated risk of obesity in adulthood (13, 14).

The Developmental Origins of Health and Disease hypothesis posits that nutritional, environmental, hormonal, and metabolic exposures during the periconceptional period, fetal life, and early infancy can modulate metabolic homeostasis and epigenetic programming, thereby influencing susceptibility to chronic diseases across the life course (15–17). Originating from David Barker’s ‘Barker hypothesis,’ this concept posits that fetal undernutrition or intrauterine growth restriction leading to low birth weight can reprogram metabolic functions, thereby increasing the risk of NCDs in later life (18). Subsequent investigations have demonstrated that maternal overnutrition, environment, and inflammation during gestation may be linked to heightened risk of NCDs in the offspring (19). Childhood obesity is considered a strong predictor of adult obesity and is linked to increased risks of early-onset NCDs (20, 21). Studies have shown that offspring born to mothers with higher pre-pregnancy body weight are likely to develop obesity by the age of nine, suggesting that maternal nutritional status may have a lasting impact on the child’s long-term risk of obesity (22). Importantly, about 15% of children aged 2 to 15 in England were classified as obese in 2022 (23). Adiposity rebound (AR) is the second rise in BMI after it reaches its lowest point in early childhood (24). This typically occurs between the ages of 2 and 8 years, after which BMI begins to increase again (25, 26). The average age of rebound typically occurs around 6 years (25). Studies have demonstrated that an earlier onset of AR, particularly before the age of 2, is related to an importantly elevated risk of metabolic disorders in adulthood (25). Therefore, the period from birth to 6 years of age may represent a critical window for the development of metabolic regulatory systems and the translation of intrauterine environmental exposures into long-term health outcomes.

The fetal period also represents a critical window for immune system development. The yolk sac is the first site of primitive hematopoiesis during embryonic development (27). By embryonic week 4, hematopoietic progenitors with stem cell-like properties emerge in both the yolk sac and the aorta-gonad-mesonephros region, giving rise to various hematopoietic lineages, including macrophages, mast cells, natural killer cell precursors, innate lymphoid cell progenitors, as well as erythroid and megakaryocytic cells (28). From gestational weeks 6 to 9, the fetal liver and subsequently the bone marrow become the principal sites of hematopoiesis, supporting the generation of a full spectrum of hematopoietic cells, including erythrocytes, megakaryocytes, myeloid cells, and lymphocytes. Pre-B cells appear by week 7, and mature B cells can be detected by week 9 (28). Between weeks 8 and 11, early lymphoid progenitors from the liver migrate to the thymus, where they differentiate into T cells under the influence of the thymic microenvironment (28). During mid-to-late gestation, neutrophils and their precursors begin to mature, and the immune system continues to develop (28). Various immune cell populations migrate to peripheral tissues, such as the skin, gut, lungs, and kidneys (28). By birth, a preliminary but functional immune network is established, providing foundational immune defense in the neonatal period (28). MO is often associated with a chronic low-grade systemic inflammatory state in mothers, which activates multiple immune and inflammation-related signaling pathways. Elevated levels of pro-inflammatory cytokines in the maternal circulation, such as TNF-α, IL-6, and IL-2, may regulate the expression of key immune-related genes in the fetus through epigenetic mechanisms (29, 30).

Although multiple immune organs, including the thymus and bone marrow, play essential roles in fetal immune development, this review did not initially focus on a single immune organ. In this systematized review, we observed that alterations in placental immune signaling consistently emerged as some of the most prominent and mechanistically informative findings across both human and animal studies. The placenta functions not only as the central organ for the exchange of nutrients and waste products between the mother and fetus, but also as a key regulator of fetal immune homeostasis (31, 32). These maternal inflammatory signals may be transmitted across the placenta and may alter fetal immune development, thereby contributing to the establishment of an immunological milieu that influences fetal developmental programming (33). In early human pregnancy, the predominant immune cells in the placenta are uterine natural killer cells and macrophages, together accounting for approximately 90% of all leukocytes (34). Around 10% of immune cells are T lymphocytes, and smaller populations of dendritic cells, mast cells, granulocytes, and B lymphocytes can also be detected (34). Toll-like receptors (TLRs) are key components of the innate immune system that recognize danger-associated molecular patterns. In the context of MO, TLR2 and TLR4 have been shown to interact with circulating free fatty acids in placental macrophages (35, 36). In MO, elevated levels of cytokines and chemokines, produced either by maternal tissues or by immune cells within the placenta, can interact with toll-like receptors (particularly TLR2 and TLR4) expressed on placental macrophages. This interaction activates intracellular signaling pathways, such as the NF-κB and p38 MAPK pathways, leading to a pro-inflammatory environment within the placenta. Chronic placental inflammation may interfere with the tightly regulated immune signaling between the mother and fetus, disrupting the development of the fetal immune system. This immune imbalance in early life may contribute to long-term changes in immune programming, potentially increasing the child’s risk of obesity, allergic conditions, and other immune-related disorders after birth.

Numerous studies have examined the impact of MO on the health of offspring. Most existing reviews focus on maternal immunometabolism problems or changes in gut microbiota. However, its impact on the offspring’s immune system has not been fully reviewed. Immune pathways that may link MO to offspring immune development are often overlooked. Current studies have paid insufficient attention to how MO affects offspring immune cell composition, cytokine secretion profiles, and inflammatory signaling pathways involved in fetal immune regulation. The immune system may serve as a crucial mediator between maternal metabolic status and long-term health outcomes in the offspring, particularly in shaping immune homeostasis and disease susceptibility. Therefore, a systematized evaluation is needed to determine whether MO reshapes fetal immune development through specific immune pathways.

## 2 Study aims and objectives

This review aims to explain how MO may affect the development of the offspring’s immune system. It focuses on changes in immune cell composition, abnormal cytokine secretion, and activation of inflammatory pathways. We conducted a systematized review to summarize and analyze the literature on the impact of MO on offspring immune development from 2010 to 2025. Specifically, it seeks to address the following questions (1): Does MO contribute to increased offspring weight? Hypothesis: We predict that MO will be related to increased offspring weight because maternal metabolic and inflammatory alterations can modify placental nutrient transport and fetal energy metabolism, thereby promoting adipose tissue deposition and weight gain. (2) What immune-related parameters are involved in this association? Hypothesis: We predict that MO will be linked with alterations in immune-related parameters because MO is accompanied by chronic low-grade inflammation, which may influence cytokine profiles and immune cell composition in the placenta and maternal circulation. (3) How is placental inflammatory signaling altered in the context of MO? Hypothesis: We predict that MO will be accompanied by altered placental signaling because obesity-related inflammation activates inflammatory signaling pathways and disturbs the balance between pro- and anti-inflammatory mediators, thereby influencing fetal immune development. These findings may guide the prevention of early-onset obesity in offspring and the reduction of adverse pregnancy outcomes.

## 3 Materials and methods

### 3.1 Search strategy

We conducted a systematized literature search to investigate the impact of MO on immune-related outcomes in offspring aged 0–6 years, as well as in relevant animal models. This review adopted a systematized review approach. Specifically, it incorporated key systematic elements, including database searches, predefined inclusion and exclusion criteria, a PRISMA flow diagram, and risk-of-bias assessment. However, it did not meet all criteria for a full systematic review (e.g., prospective registration and dual independent screening).

Given the substantial variation across included studies in species, animal models, dietary interventions, and measured immune outcomes, formal meta-analysis was not considered appropriate. Instead, findings were synthesized narratively. The detailed methodology is described below.

**Figure 1:**
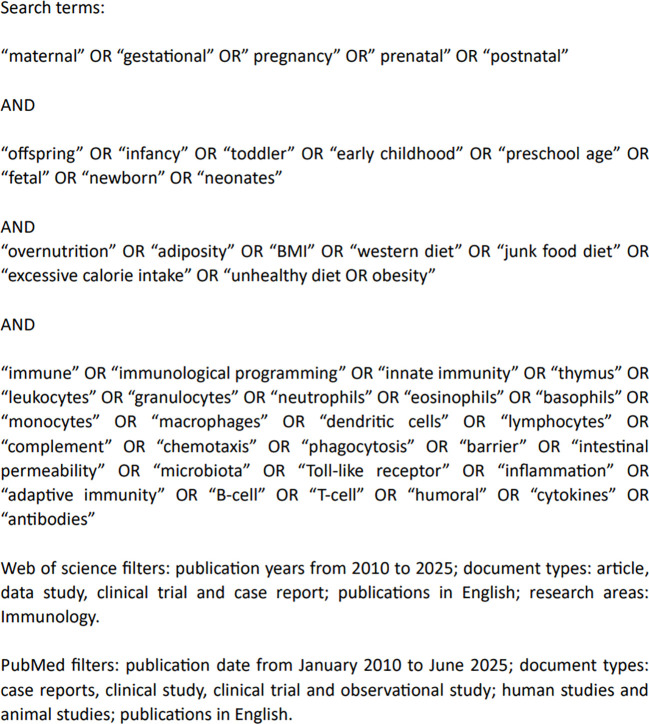
Full search strategy in PubMed, including search terms and filters.

The search was performed in the PubMed and Web of Science databases, covering the period from January 2010 to June 2025. The search strategy was constructed using Boolean operators (AND, OR) to combine keywords across four core conceptual domains: (1) maternal-related terms, (2) offspring developmental stages, (3) obesity and nutritional factors, and (4) immune system terms. Only articles published in English were included, encompassing case reports, clinical trials, observational studies, and experimental studies involving humans or animals. The scope was limited to research within the field of immunology. The detailed search terms are provided in **Figure 1**.

### 3.2 Inclusion and exclusion criteria

#### 3.2.1 The following studies were included

- Both human and animal studies were eligible for inclusion.
- Human studies involving children aged 0–6years.
- Diet-induced maternal obesity (BMI ≥30 kg/m²) occurring pre-pregnancy or during the first trimester (≤12 weeks of gestation).
- Studies must assess obesity-related immune outcomes in offspring and/or placenta or cord blood, including inflammatory cytokines, immune cell profiles, or epigenetic regulation.
- Only articles published in English were considered.
- Only peer-reviewed journal articles or scholarly book chapters were included.

#### 3.2.2 The following studies were excluded

- Non-original research articles (i.e. review articles, opinion pieces, commentaries, conference, abstracts, and study protocols).
- Studies without full-text access or available only as preprints.
- No offspring immune analysis.
- Studies involving maternal complications during pregnancy.
- Studies using genetically modified maternal models.
- Animal studies involving maternal medication interventions.
- Animal studies involving maternal exposure to chemical or biological factors.
- Animal studies involving maternal infections.
- Animal studies involving maternal diet (i.e. fructooligosaccharide) or exercise interventions during experiment.
- No weight or BMI data on mothers.
- Studies in which offspring immunity indicators were not analyzed separately for obese versus non-obese groups.
- Studies that did not report offspring outcomes separately for obese versus non-obese mothers.

### 3.2 Literature selection process

The systematized review imported the retrieved records into EndNote and Rayyan for duplicate removal following completion of the database search. Rayyan was selected as the primary tool for subsequent screening after assessing the accuracy of deduplication. Predefined inclusion and exclusion criteria were applied in 3 screening phases. During the initial screening phase, review articles, opinion pieces, commentaries, conference abstracts, study protocols, publications without full-text availability, and studies that did not assess offspring immune outcomes were excluded because they do not provide primary data and often lack sufficient methodological detail for quality assessment, making it impossible to determine whether their experimental design and procedures meet the inclusion criteria of this study.

In the second screening stage, studies involving maternal pregnancy complications (e.g., gestational diabetes mellitus and preeclampsia) were excluded, as these conditions independently alter inflammatory and metabolic pathways that influence fetal immune development and growth, thereby introducing clinical and immunological confounding and potentially obscuring effects attributable to MO per se. Animal model studies involving exposure to medicines, chemical agents, or biological agents (e.g., zearalenone, lipopolysaccharide, and allergens), as well as genetic modifications (e.g., NG2 ablation, non-obese diabetic mice, and Iga^−/−^ [Igha^tm1Grh^] mice), were excluded. Mothers living with obesity and maternal models infected with viral or parasitic infections were also excluded. Because such factors may introduce confounding effects unrelated to high-fat diet (HFD)-induced obesity and complicate the interpretation of offspring immune changes. Non-HFD models were excluded due to differing metabolic characteristics from the main pathophysiological mechanisms of maternal obesity. Likewise, studies involving exercise or dietary interventions were excluded, as such interventions may alter maternal inflammatory and metabolic states, potentially masking the original exposure effects. Finally, studies without maternal obesity–related data were excluded, as they could not support correlation analyses between maternal obesity status and immune changes.

In the final screening phase, studies where offspring were older than six years were excluded, as the review focused on immune development in early life. Studies that did not report offspring weight data were also excluded, since this information is necessary to examine links between maternal obesity and offspring growth or immune outcomes. Studies with maternal BMI below 30 kg/m² were excluded because they did not meet our definition of obesity. Similarly, studies that failed to distinguish obese from non-obese maternal groups clearly or did not stratify immune outcomes by maternal obesity status were excluded, as these limitations prevent meaningful comparisons. Finally, we excluded studies without maternal weight data from the pre-pregnancy or first-trimester period (≤12 weeks) (37), because missing information from this key exposure window would reduce the reliability of the findings.

In the case of one study with ambiguous maternal BMI data, we reached out to the corresponding author via email and obtained clarification, ensuring the accuracy and completeness of the information included in the review. All screening procedures were conducted by the first author. Any uncertainties were resolved through discussion and consensus within the author team.

### 3.3 Data extraction and synthesis

The data from the included studies were extracted and organized using Excel spreadsheets. For human observational studies, the extracted information included: first author and year of publication, study location, sample size, maternal pre-pregnancy or first-trimester BMI, offspring characteristics (e.g., age, birth weight), sample sources, and control group details.

For animal studies, the extracted data included: first author and year of publication, type of animal model, experimental subjects, control group information, age of offspring, and the type of dietary model used during feeding.

### 3.4 Methodological quality assessment

To evaluate the quality of the included studies, this review applied distinct tools to assess the risk of bias and strength of evidence in animal and human studies, respectively. All assessments were conducted independently by two reviewers. Any disagreements were resolved through discussion until consensus was reached.

For animal studies, the SYRCLE’s Risk of Bias Tool (Systematic Review Centre for Laboratory Animal Experimentation) was used. This tool comprises ten domains covering sequence generation, baseline characteristics, allocation concealment, random housing, blinding of personnel, random outcome assessment, blinding of outcome assessment, incomplete outcome data, selective outcome reporting, and other sources of bias. Each domain was rated as low risk, high risk, or unclear risk based on the reporting quality of each study.

For human studies, the internationally recognized LEGEND (Let Evidence Guide Every New Decision) evidence assessment framework was adopted. The LEGEND tool evaluates aspects such as validity, reliability, and applicability, and categorizes the overall strength of evidence into a good quality cross-sectional study (4a), a lesser quality cross-sectional study (4b), a good quality prospective cohort study (3a), a lesser quality prospective cohort study (3b), or not valid, reliable, or applicable. These ratings may help guide the integration of evidence and the interpretation of the reliability of the results.

## 4 Results

### 4.1 Literature search results

**Figure 2:**
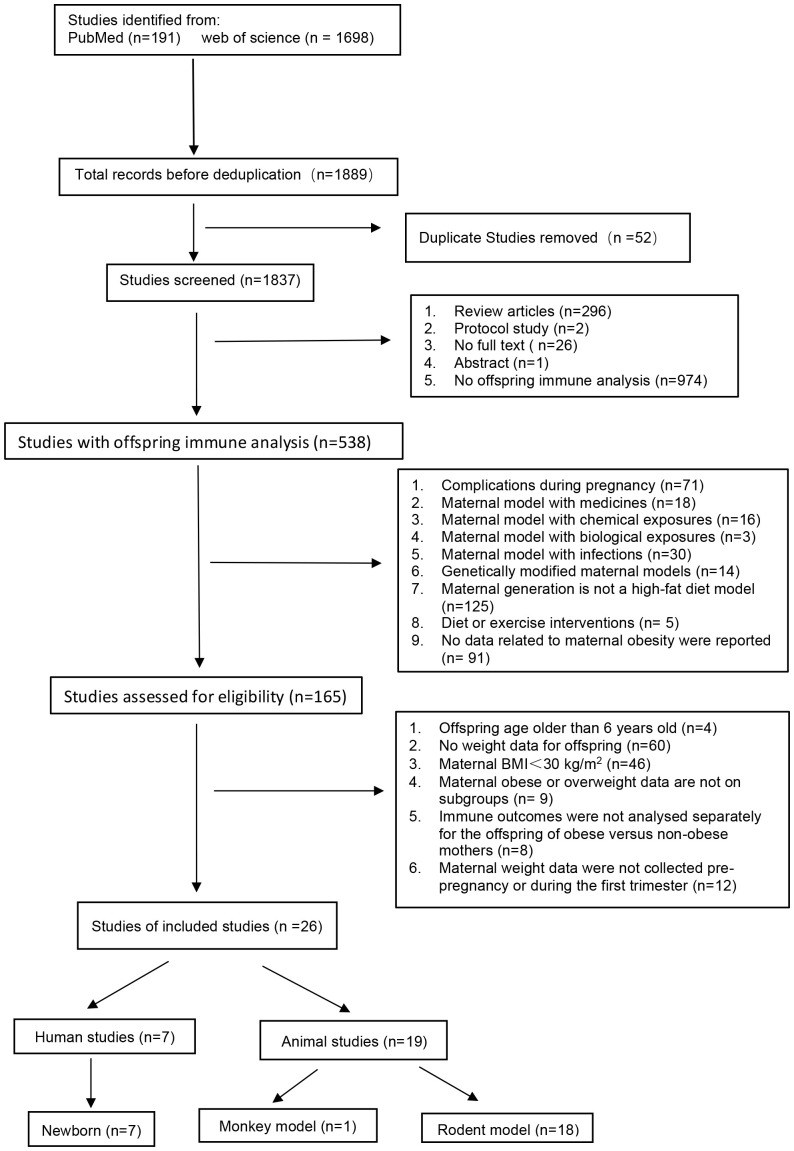
PRISMA diagram.

1,889 records were retrieved in total from the PubMed and Web of Science databases. After removing duplicates, 1,837 records remained. Based on the predefined inclusion and exclusion criteria, 1,809 records were excluded. Ultimately, 26 studies were included in the review, comprising 7 human observational studies and 19 animal experimental studies. The detailed study selection process is shown in the PRISMA flow diagram (**Figure 2**).

### 4.2 Characteristics of articles included in systematized review

This review included seven human observational studies published between 2010 and 2023. For human studies, six cross-sectional studies and one prospective cohort study were included. Studies were conducted in diverse geographical locations, including Spain, Sweden, the United States, Mexico, and the United Kingdom. Maternal sample sizes varied considerably, with the number of participants in the obese group ranging from as few as 5 to over 800, depending on BMI stratification. Maternal obesity was generally defined based on pre-pregnancy or early pregnancy BMI, most commonly using a threshold of ≥30 kg/m², with some studies further stratifying obesity levels (e.g., 30–34.9 kg/m² and >35 kg/m²).

Offspring characteristics included birth weight and age at sampling, although not all studies provided complete information. Biological samples used for immune profiling were diverse and included placental tissue, umbilical cord blood, dried blood spots, and newborn peripheral blood collected after birth (**Table 1**).

**Table 1:** Characteristics of included human observational studies of MO during pregnancy.

Author, year	Publication	Country	Maternal obese sample size (n)	Maternal pre-pregnancy or first trimester BMI (kg/m²) on obese group	Maternal pre-pregnancy or first trimester BMI (kg/m²) on non- obese group	Maternal non-obese sample size (n)	Maternal Age (years)	Maternal obese group offspring sample size (n)	Maternal non -obese group offspring sample size (n)	Specimen source	Maternal obese group birth weight (g)	Maternal non -obese group birth weight (g)
Altmäe, (38)	PLOS one	Spain	5	33.6 ± 2.0^e^	21.0 ± 2.0^e^	5	25-35	5	5	Placenta	3602 ± 595^e^	3344 ± 302^e^
Allbrand, 2022 (39)	Placenta	Sweden	109	≥35	Not reported	Not reported	18-45	109	Not reported	Placenta	3894 ± 492	Not reported
Broadney, (40)	International journal of obesity	United States	BMI 30–34.9 (n=373); BMI >35.0 (n=380)	≥30	Normal weight: 18.5-24.9; Underweight: <18.5; Overweight: 25-29.9	Normal weight (n=1384); Underweight (n=64); Overweight (n=773)	BMI (30-34.9) 30.9 (5.8) BMI (>35.0) 30.2(6.0)^b^	BMI 30-34.9 (n=373) BMI >35.0 (n=380)	Normal weight (n=1384); Underweight (n=64); Overweight (n=773)	Dried blood spots	BMI (30-34.9) 3265.7 (633.7) BMI (>35.0) 3252.3 (765.4)^b^	Normal weight: 3153.2 (655.3); Underweight: 2947.2 (747.7); Overweight: 3237.7 (680.8)^b^
Aye, (41)	Biology of reproduction	United States	24	34.8 ± 1.2^c^	Normal weight: 25; Overweight: 25-30	Normal (n=23); Overweight (n=13)	18-24	24	Normal weight: n=23; Overweight: n=13	Venous cord blood; Placenta	3457 ± 50^c^	Normal weight: 3238 ± 50; Overweight: 3329 ± 93^c^
Shook, (42)	Placenta	United States	15	35.6 (32.5-39.2)^d^	BMI <30	19	29.0-32.5	15	19	Placenta	3600 (3200-4100)^d^	3400 (3300-3600)^d^
Méndez-Mancilla, (43)	Pediatric obesity	Mexico	10	34.55 ± 3.92^e^	Normal weight: 21.92 ± 1.63; Overweight: 27.54 ± 1.42^e^	Normal weight: n=21; Overweight: n=10	18-40	10	Normal weight: n=21; Overweight: n=10	Peripheral blood	3306 ± 328.9^e^	Normal weight; 3200 ± 332.5; Overweight; 3116 ± 271.8^e^
Roberts, (44)	Placenta	United Kingdom	33	39.9 ± 4.4-42.7 ± 4.3^e^	20-25	47	33.6 ± 4.5^e^	Not reported	Not reported	Peripheral blood; Placenta	3548 ± 527.9 3617.9 ± 471.0^e^	3493.2 ± 480.3- 3498.7 ± 485.6^e^

BMI, body mass index; b, mean (standard deviation); c, mean ± SEM; d, median (IQR); e, mean ± SD.

Nineteen animal studies published between 2010 and 2024 were included. The majority use*d C57BL/6J* mice (*n* = 12) as the model organism. Other models included *Wistar* rats (*n* = 2), *Sprague–Dawley* rats (*n* = 2), *Long–Evans* rats (*n* = 1), *Swiss* mice (*n* = 1), and *rhesus* macaques (*n* = 1). Control groups were typically fed a normal or low-fat diet. Most studies focused on first-generation (F1) offspring, and only two extended observations to the second generation (F2). Tissue collection in rodent offspring was commonly performed during mid-to-late gestation (e.g., fetal day 14.5–21) or between 1 and 3 months after birth. In some rodent studies (*n* = 8), sampling was extended to postnatal day (PD) 90 or adulthood. Biological samples collected from offspring included placenta, liver, adipose tissue, brain, and blood (**Table 2**).

**Table 2:** Characteristics of included animal studies of diet-induced obesity during pregnancy.

Author, year	Publication	Animal model	Research offspring generation	Control group?	Offspring specimen source	Offspring age (as reported)	Offspring feeding
Ding, (45)	International journal of obesity	C57BL/6J mice	HFD F2	Yes; HFD F0, HFD F1 and, control standard diet	Visceral parametrial adipose tissue	9 months	HFD
Wallace, (46)	Scientific reports	C57BL/6J mice	HFD F1	Yes; control standard diet	Placenta	Day 14.5 of pregnancy	No feeding
Nash, (47)	Cell reports	Rhesus macaque	WSD F1	Yes; control standard diet	Bone marrow cells; liver	Sample1; Day 130–135 of pregnancy; Sample2; 3 years old	7–8 months HFD, 8 months-3 years old normal diet
Chang, 2019	Scientific reports	C57BL/6J mice	HFD F1	Yes; P/G	Adipose tissue	24 weeks	12 weeks normal diet 12–24 weeks HFD
Matuszewska, (48)	Scientific reports	Wistar rats	CAF F1	Yes; normal diet	Trunk blood	PD25	No feeding
Gohir, (49)	The journal of physiology	C57BL/6J mice	HFD F1	Yes; normal diet	Intestines; placenta	Day of 18.5 pregnancy	No feeding
Alfaradhi, (50)	Endocrinology	C57BL/6J mice	HFD F1	Yes; normal diet	Epididymal adipose tissue	8 weeks	PD 21–8 weeks normal diet
Buckley, (51)	Biology	C57BL/6J mice	HFD F1	Yes; normal diet	Truncal blood; whole hypothalamus	PD 9 and 4 months	PD 21–4 months normal diet
Murabayashi, (52)	European journal of obstetrics & gynecology and reproductive biology	C57BL/6J mice	HFD F1	Yes; normal diet	Male; subcutaneous abdominal adipose tissue	Day 17 of pregnancy	No feeding
Candia, (53)	Antioxidants	C57BL/6J mice	HFHS F2	Yes; normal diet	Placenta; blood	Day 17.5 of pregnancy	No feeding
Crew, (54)	Placenta	Nulliparous albino Wistar rats	CAF F1	Yes; normal diet	Placenta; truncal blood	Day 21 of pregnancy	No feeding
Winther, (55)	Neuroscience	Sprague-Dawley rats	HFD F1	Yes; normal diet	Hippocampus	PD91	Normal diet
Liang, (56)	The journal of physiology	C57BL/6J mice	HFDF1	Yes; normal diet	Epididymal white adipose tissue of male offspring	3 months	Obese offspring; 1. HFD 2. normal diet
Sasaki, (57)	Neuroscience	Long Evans rats	HFD F1	Yes; normal diet	Brain	PD90	PD 21–90 normal diet
Payolla, (58)	Molecular and cellular endocrinology	Swiss mice	HFD F1	Yes; normal diet	Male; blood; epididymal fat pad; WAT; liver	PD28	Day 18–28 normal diet
e-Lacerda; (59)	Nutrients	C57BL/6J mice	HFD F1	Yes; normal diet	Lung	8 weeks	Normal diet
Edlow, (60)	international journal of developmental neuroscience	C57BL/6J mice	HFD F1	Yes; normal diet	Brain; placenta	Day of 17.5 pregnancy	No feeding
Bilbo, (61)	The FASEB journal	Sprague-Dawley rats	SFD; TFD F1	Yes; LFD	Brain; liver; blood; tissue	PD 85-95	Normal diet
Liu, (62)	Molecular medicine reports	C57BL/6J mice	MHFD F1	Yes; normal diet	Tissue; blood	24 weeks	MHFD

HFD, high-fat diet; Three generations (F0, F1 and F2); WSD, Western-style diet; CAF, cafeteria diet; HFHS, high-fat/high-sugar diet; SFD, high saturated-fat diet; TFD, high trans-fat diet; LFD, low-fat diet; MHFD, moderate high-fat diet; PD, postnatal day.

### 4.3 Offspring birth weight outcomes

Birth weight outcomes were reported in 15 of the 26 included studies (57.7%), comprising six human observational studies and nine animal studies, all of which compared birth outcomes between MO and NMO groups. All offspring in these studies were newborns, and no data were available for early childhood (ages 1–6 years). Statistical comparison of birth weight between MO and NMO groups was available from 14 studies; one study (Roberts et al., 44) did not report a *p-*value. Across these studies, higher offspring birth weights in the MO group were reported in three studies (11.5%), including two human studies and one animal study. Eight studies (30.7%) reported no significant difference in birth weight between the MO and NMO groups, whereas three studies (11.5%) observed significantly lower birth weights in offspring born to mothers with obesity compared with controls (**Table 3**).

**Table 3:** Birth weight differences in human and animal studies between offspring of MO and NMO.

MO versus NMO on offspring birth weight	Number of papers^a^	Statistically significant?	References
MO > NMO	9	5 NS; 3 Yes; 1 Not reported	1. Broadney et al., 2017 (40); Aye et al., 2014 (41); Liang et al., 2016 (56) (Yes); 2. Altmäe et al., 2017 (38); Shook et al., 2023 (42); Méndez-Mancilla et al., 2018 (43); Wallace et al., 2019 (46); Edlow et al., 2020 (60) (NS); 3. Roberts et al., 2011 (44) (p-value not reported).
MO ≈ NMO	3	all NS	Nash et al., 2023 (47); Murabayashi et al., 2013 (52); Gohir et al., 2019 (49).
MO < NMO	3	all Yes	Candia et al., 2024 (53); Crew et al., 2016 (54); Payolla et al., 2016 (58).
Total	15		

NS, statistically non-significant difference between groups; Yes, statistically significant (p<0.05); a, Fetal weight was reported in 15 of 26 papers.

### 4.4 Summary of offspring immune alterations

To investigate the impact of MO on the offspring’s immune system, immune outcomes from both human observational studies and animal experiments were reviewed. Due to the large number and heterogeneity of immune read-outs reported in animal studies, we focused on immune parameters that were assessed in two or more studies to enhance comparability and robustness of the synthesis. Accordingly, the analysis centers on alterations in immune cell populations, cytokines, and key inflammatory markers and pathways (**Tables 4**, **5**).

**Table 4:** Summary of offspring immune alterations in human studies.

Immune parameter	Number of studies	Specimen source	Immune alterations in offspring of mothers with obesity compared to those without obesity	References
Gene	1	Placenta	CCL2; HLA-DRB1; AREG↑; IL1R2; PLA2G7; LAIR2; MMP12; PRG2 ↓	Altmäe et al., 2017 (38)
Neutrophil	1	Placenta	Number of neutrophils ↑	Roberts et al., 2011 (44)
MicroRNA	1	Peripheral blood	MiR-155; miR-181a; miR-221 ↓	Méndez-Mancilla et al., 2018 (43)
Macrophage	1	Placenta	CD163 + HBCs density ↑	Shook et al., 2023 (42)
Antibody	1	Dried blood spots	IgM ↑, IgG1, IG4 ↓	Broadney et al., 2017 (40)
CRP	1	Dried blood spots	CRP ↑	Broadney et al., 2017 (40)
MCP-1	1	Placenta	MCP-1 ↑	Roberts et al., 2011 (44)
CXCR2	1	Placenta	CXCR2 ↑	Roberts et al., 2011 (44)
IL-8	1	Placenta	IL-8 ↑	Roberts et al., 2011 (44)
IL-6	1	Dried blood spots	IL6 ↓	Méndez-Mancilla et al., 2018 (43)
Inflammatory Pathways	1	Placenta	1. PHT cultured: MCP-1 activate the P-p38-MAPK; TNFα Activate the P- p38-MAPKP; Y705 P-STAT3 and STAT3 total amount. 2.Maternal BMI ↑ P-p38 MAPK (Y812/Y180); P-STAT3(Y750) ↑. 3. Fetal weight ↑P-p38 MAPK(Y812/Y180) ↑.	Aye et al., 2014 (41)

↑= higher in offspring of mothers with obesity than in offspring of mothers without obesity; ↓= lower in offspring of mothers with obesity than in offspring of mothers without obesity; (number) = numbers of papers.

**Table 5:** Summary of offspring immune alterations in animal studies.

Immune parameter	Number of studies	Time points for offspring sample testing	Trend of change in offspring of MO compared with NMO	Specimen source	References
TNF-α	11	PD25; PD28; 6 weeks; 8 weeks; 4 months; E17.5; E17; E18.5; PD91; 24 weeks; 16 weeks; 3 months	↑ (11); ↓ (1)	Blood	Candia et al., 2024 (53); ↑ Payolla et al., 2016 (58); ↑ Liu et al., 2018 (62); ↑ Matuszewska et al., 2021 (48); ↑
				Lung	e-Lacerda et al., 2019 (59); ↑
				Adipose tissue	Alfaradhi et al., 2016 (50); ↑ Murabayashi et al., 2013 (52); ↑ Liang et al., 2016 (56); ↑
				Hypothalamus	Buckley et al., 2024 (51); ↓
				Brain	Winther et al., 2018 (55); ↑ Edlow et al., 2019 (60); ↑
				Placenta	Edlow et al., 2019 (60); ↑
IL-6	10	E14.5; E130-135; PD25; 4 months; E18.5; PD85-95; E21; 3 months; PD90	↑ (8); ↓ (2)	Placenta	Wallace et al., 2019 (46); ↑ Crew et al., 2016 (54); ↑
				HSPC	Nash et al., 2023 (47); ↑
				Blood	Matuszewska et al., 2021 (48); ↑ Bilbo et al., 2010 (61); ↑ Buckley et al., 2024 (51); ↓ Candia et al., 2024 (53); ↑
				Adipose tissue	Liang et al., 2016 (56); ↑
				Hypothalamus	Buckley et al., 2024 (51); ↓
				Amygdala	Sasaki et al., 2013 (57); ↑
IL-10	5	3 years old; PD25; E14.5; PD28; 24 weeks	↑ (3); ↓ (3)	BMDM	Nash et al., 2023^f^ (47);↓ Nash et al., 2023^f^ (47);↑
				Blood	Matuszewska et al., 2021 (48); ↑ Liu et al., 2018 (62); ↓
				Adipose tissue	Payolla et al., 2016 (58); ↓
				Placenta	Wallace et al., 2019 (46); ↑
MCP-1 (CCL2)	4	E14.5; 8 weeks; PD91; 3 months	↑ (4)	Placenta	Wallace et al., 2019 (46); ↑
				Adipose tissue	Liang et al., 2016 (56); ↑ Alfaradhi et al., 2016 (50); ↑
				Brain	Winther et al., 2018 (55); ↑
NF-kB	4	3 years old; E18.5; PD90; E14.5	↑ (4)	Small intestine	Gohir et al., 2019 (49); ↑
				BMDM	Nash et al., 2023 (47); ↑
				Amygdala	Sasaki et al., 2013 (57); ↑
				Placenta	Wallace et al., 2019 (46); ↑
TNF	3	9 months; E18.5; 3 years old	↑ (4)	Adipose tissue	Ding et al., 2014 (45); ↑
				Placenta	Wallace et al., 2019 (46); ↑
				BMDM	Nash et al., 2023 (47); ↑
				HSPC	
TLR4	3	9 months; E18.5; E21	↑ (2); ↓ (1)	Adipose tissue	Ding et al., 2014 (45); ↑
				Small intestine	Gohir et al., 2019 (49); ↑
				Placenta	Crew et al., 2016 (54); ↓
ARG1	3	E14.5; E18.5; PD28	↓ (3)	Placenta	Gohir et al., 2019 (49);↓ Wallace et al., 2020 (46); ↓
				Adipose tissue	Payolla et al., 2016 (58); ↓
STAT3	2	3 years old; E18.5	↑ (1); ↓ (1)	BMDM	Nash et al., 2023 (47); ↑
				Placenta	Gohir et al., 2019 (49); ↓
VEGF	2	E14.5; E18.5	↑ (2)	Placenta	Gohir et al., 2019 (49); ↑ Wallace et al., 2019 (46); ↑
Traf6	2	E14.5; E18.5	↑ (1); ↓ (1)	Placenta	Gohir et al., 2019 (49); ↓ Wallace et al., 2020 (46); ↑
TLR2	2	9 months; E14.5	↑ (2)	Adipose tissue	Ding et al., 2014 (45); ↑
				Placenta	Wallace et al., 2019 (46); ↑
TLR1	2	9 months; 3 years old	↑ (2)	Adipose tissue	Ding et al., 2014 (45); ↑
				BMDM	Nash et al., 2023 (47); ↑

↑= higher in maternal obese than maternal non-obese offspring; ↓= lower in maternal obese offspring than maternal non-obese; Embryonic day (E); Postal Day (PD); Bone marrow-derived macrophage (BMDM); Hematopoietic stem and progenitor cell (HSPC); ^f^= IL-4 stimulate, LPS stimulate or LPS combined with IFN-γ.

#### 4.4.1 Immune outcomes in human studies

In the study by Altmäe et al., placental genes such as C-C motif chemokine ligand 2 (CCL2), major histocompatibility complex, class II, DR Beta 1(HLA-DRB1, and amphiregulin(AREG)mRNA (messenger RNA) expression were found to be upregulated, whereas Interleukin 1 receptor type 2(IL1R2), phospholipase A2 group VII(PLA2G7), leukocyte-associated immunoglobulin-like receptor 2(LAIR2), matrix metallopeptidase 12(MMP12), and proteoglycan 2(PRG2)mRNA expression were downregulated. These genes are all involved in inflammatory processes and immune dysregulation (38).

Interleukin-1 beta(IL-1β), a potent pro-inflammatory cytokine belonging to the IL-1 family, is primarily secreted by monocytes and macrophages (63). Elevated IL-1β protein and mRNA expression in placental tissues of offspring born to MO versus NMO were reported in two (7.7%) human studies (41, 44). Interleukin 1 receptor type 2 (IL-1R2) is constitutively expressed on B cells, neutrophils, monocytes, and macrophages. Acting as a decoy receptor, IL-1R2 prevents the binding of IL-1β to its signaling receptor IL-1R1, thereby attenuating downstream inflammatory signaling pathways (64). Downregulation of IL-1R2 is suggested to permit activation of IL-1β–mediated inflammatory pathways in the placenta (38). CCL2, also referred to as monocyte chemoattractant protein-1 (MCP-1), is a pivotal chemokine involved in the recruitment of monocytes and macrophages (38, 44, 65). Upregulated mRNA expression of CCL2 along with enhanced CD163^+^ Hofbauer cells (HBCs) infiltration, while the expression level of the associated MMP12 is notably downregulated in the placenta, and elevated levels of C-reactive protein (CRP) were also observed in dried blood spots (38, 40, 42). These findings suggest the presence of placental inflammation in MO.

Interleukin-8 (IL-8) is a key chemoattractant for neutrophils. Placental IL-8 and its receptor C-X-C motif chemokine receptor 2 (CXCR2) mRNA levels were higher in offspring of MO than in those of NMO, and this was accompanied by higher neutrophil counts in the placenta (44).

Interleukin-6 (IL-6) exhibits context-dependent dual functions, acting as either a pro-inflammatory or anti-inflammatory mediator under different conditions, and can be secreted by various tissues and cell types (66). Its expression is mainly induced by IL-1β and tumor necrosis factor (TNF) and is further regulated by TLR signaling, adipokines, and other cytokines (66). Experimental studies have shown that IL-6 deficiency increases susceptibility to obesity in adulthood (67). Méndez-Mancilla et al. showed that dried blood spots analysis revealed lower circulating IL-6 levels in offspring of MO compared with those of NMO (43).

In the study by Aye et al., experiments using primary human trophoblast (PHT) cultures showed that MCP-1 activated the phosphorylation of p38 mitogen-activated protein kinase (P-p38-MAPK), whereas TNF-α not only activated P-p38-MAPK but also promoted signal transducer and activator of transcription 3 phosphorylation at Y705 (P-STAT3) and increased total STAT3 protein levels. Furthermore, maternal BMI was positively associated with higher placental levels of P-p38 MAPK at Y812/Y180 and P-STAT3 at Y750, while fetal weight was similarly correlated with elevated P-p38 MAPK at Y812/Y180. These findings suggest that the activity of placental p38-MAPK signaling pathways may be enhanced in MO, potentially promoting inflammatory responses, which could in turn influence fetal immune development and weight gain.

Immunoglobulin M (IgM) is the first antibody synthesized and secreted by B cells in response to initial antigen stimulation, and it facilitates the phagocytosis of apoptotic cells (68). The clearance of apoptotic cells contributes to the formation of an anti-inflammatory microenvironment, inducing B cells and macrophages to secrete higher levels of interleukin-10 (IL-10). During normal pregnancy, immunoglobulin G (IgG) is the unique antibody class capable of passing through the placental barrier to enter the fetal circulation (40). Altmäe et al. identified IgG as an upstream activator of multiple dysregulated genes, such as CCL2, in the placenta of MO. In the study by Broadney et al., offspring of MO had higher serum IgM levels and lower IgG1 and IgG4 levels compared with those of mothers with a normal body weight. Higher IgM levels may be linked to obesity-induced fetal oxidative stress (38).

MicroRNAs (miRNAs) are a class of small endogenous RNAs, approximately 22 nucleotides long, that lack the capacity to code for proteins. They constitute a highly conserved family of transcripts, accounting for less than 0.02% of the total cellular RNA content (69). They represent the most well-characterized small ncRNA class and are estimated to impact over 60% of protein-coding genes. In the study by Méndez-Mancilla et al., serum concentrations of miR-155, miR-181a, and miR-221 were significantly lower in the offspring of MO compared with those of NMO. Furthermore, miR-155, miR-181a, and miR-221 were found to share multiple target genes involved in immunometabolism pathways, including the B cell receptor, T cell receptor, and MAPK signaling pathways (43). In addition, several target genes within the insulin signaling pathway were also regulated by these miRNAs (43). MO may lead to dysregulation of miRNA expression in the offspring; however, the specific mechanisms involved remain unclear.

#### 4.4.2 Immune outcomes in animal studies

Evidence from animal studies indicates that MO induces immune alterations in offspring across multiple organs and biological compartments, including the systemic circulation, organs, placenta, and immune cell–associated compartments. Accordingly, immune outcomes in this section are summarized by tissue and biological compartment (**Table 5**).

##### 4.4.2.1 Systemic and circulating immune compartments

At the systemic level, several studies assessed inflammatory cytokines in circulating blood. In addition, immune outcomes were examined in immune cell-associated compartments, including hematopoietic stem and progenitor cells (HSPCs) and bone marrow–derived macrophages (BMDMs). TNF-α, a cytokine closely linked to metabolic disease, was the most consistently reported marker (70). Among the included studies, four (15.4%) reported higher circulating TNF-α levels in offspring of MO compared with controls. In contrast, findings for IL-6 were less consistent: three (11.5%) studies reported higher circulating IL-6 levels in offspring of MO, whereas one (3.8%) study observed lower levels.

IL-10 is an anti-inflammatory cytokine that plays a critical role in limiting excessive inflammatory responses, showed heterogeneous changes (71). Across the included studies, two (7.7%) investigations assessed circulating IL-10 levels and reported inconsistent changes, with one (3.8%) study showing higher levels and another reporting lower levels in offspring of MO.

One (3.8%) study further focused on immune cell–associated compartments, including BMDMs and HSPCs. Nash et al. (47) reported that in HSPCs, expression of IL-6, TNF was consistently upregulated, suggesting that pro-inflammatory signaling may be activated at the level of immune cell precursors. In contrast, the same study demonstrated stimulus-dependent responses in BMDMs derived from 3-year-old rhesus macaques. Following IL-4 stimulation, IL-10 expression was upregulated, whereas under lipopolysaccharide (LPS) stimulation, IL-10 expression was markedly downregulated. In addition, TLR1, TNF, NF-κB, and STAT3-related signaling pathways were overall upregulated in BMDMs.

##### 4.4.2.2 Fetal brain

Multiple studies have reported that MO is associated with altered expression of inflammatory mediators in the offspring central nervous system, affecting both the whole brain and specific brain regions, including the hypothalamus and amygdala. At the whole-brain level, two (7.7%) studies observed upregulated TNF-α expression, while another study reported upregulated MCP-1 (CCL2) expression. In contrast, immune alterations in the hypothalamus showed an opposite pattern, with one (3.8%) study reporting downregulated TNF-α expression and another observing downregulated IL-6 expression. In the amygdala, one (3.8%) study reported upregulated IL-6 expression, whereas another study identified upregulation of the NF-κB signaling pathway. Overall, these findings suggest that MO may influence offspring central nervous system development and function through brain-region–specific neuroimmune regulatory mechanisms.

##### 4.4.2.3 Fetal lung

Only one (3.8%) study reported upregulated TNF-α expression in fetal lung tissue.

##### 4.4.2.4 Fetal intestinal mucosa

Two (7.7%) studies have reported immune alterations in the fetal small intestine, with one (3.8%) study observing upregulation of NF-κB expression and another reporting upregulated TLR4 expression.

##### 4.4.2.5 Fetal adipose tissue

Multiple studies have reported immune alterations in fetal adipose tissue. Specifically, three (11.5%) studies observed upregulated TNF-α expression, while one (3.8%) study reported upregulated TNF expression. In addition, IL-6 expression was upregulated in one (3.8%) study, whereas IL-10 expression was downregulated in another. The chemokine MCP-1 (CCL2) was reported to be upregulated in two (7.7%) studies. With respect to innate immune signaling molecules, TLR1, TLR2 and TLR4 expression were each reported to be upregulated in one (3.8%) study. Notably, ARG1 expression was also reported to be upregulated in one (3.8%) study.

##### 4.4.2.6 Placenta

Multiple studies have reported placental immune alterations associated with MO. With respect to inflammatory mediators, one (3.8%) study observed upregulated TNF-α expression, two (7.7%) studies reported upregulated IL-6 expression, and one (3.8%) study reported upregulation of IL-10. In addition, upregulated expression or activity of MCP-1 (CCL2), TNF, and NF-κB was each reported in one (3.8%) study.

Regarding innate immune-related receptors, TLR2 expression was reported to be upregulated in one (3.8%) study, whereas TLR4 expression was downregulated in one (3.8%) study.

Among immune regulatory and signaling molecules, STAT3 expression was reported to be downregulated in one (3.8%) study. STAT3 is a transcription factor with dual roles, involved in both pro-inflammatory responses and immune suppression, primarily activated by cytokines such as IL-6 and IL-10 (72). Findings related to TNF Receptor-Associated Factor 6 (TRAF6) were inconsistent across studies, with one (3.8%) study reporting upregulated expression and another study observing downregulated expression. TRAF6 is a signaling adaptor protein and an E3 ubiquitin ligase that indirectly regulates gene expression by activating downstream transcription factors such as NF-κB (73).

In contrast, arginase 1 (ARG1) expression was downregulated in two (7.7%) studies. ARG1 is an essential component of the urea cycle that catalyzes the degradation of arginine to ornithine and urea (74). ARG1 plays a critical role in fetal growth and development by promoting placental angiogenesis, enhancing placental blood circulation, and increasing maternal–fetal nutrient transfer (74). Deficiency of ARG1, resulting in abnormal accumulation of arginine, can adversely affect fetal development (74). Consistently, vascular endothelial growth factor (VEGF) was reported to be upregulated in two (7.7%) studies. VEGF is a key regulator of angiogenesis and vasculogenesis during placental development (75). Together, these findings suggest that MO may influence placental angiogenesis and function in offspring through coordinated alterations in VEGF expression and ARG1-associated arginine metabolism.

### 4.5 Immune alterations in the placenta

In summarizing the impact of MO on immune alterations in offspring, immune changes were found to be most consistently observed in placental tissues. Accordingly, we summarized and synthesized findings from both human and animal studies.

In human studies, a total of five (19.2%) investigations assessed changes in placental immune parameters at the mRNA expression level. The results showed mRNA expression upregulation of IL-1β, MCP-1, CXCR2, and IL-8, and mRNA expression downregulation of IL-6. An increased abundance of CD163^+^ macrophages and neutrophils were observed, along with mRNA expression upregulation of p38-MAPK and STAT3. In addition, CCL2, HLA-DRB1, and AREG mRNA expression were upregulated, whereas IL1R2, PLA2G7, LAIR2, MMP12, and PRG2 mRNA expression were downregulated.

In animal studies, a total of five (19.2%) investigations evaluated the effects of MO on placental immune parameters in the offspring. The findings demonstrated significant alterations in the mRNA expression of various inflammation-related cytokines and signaling molecules in the placenta: IL-6, IL-10, TNF-α/TNF, MCP-1 (CCl2), NF-κB, TLR2, IL-1β, ARG1, and VEGF mRNA expression were upregulated; STAT3 mRNA expression was downregulated; and TRAF6 showed inconsistent trends, with both upregulation and downregulation reported across studies. These results suggest that MO may induce placental inflammation in the offspring through activation of inflammation-related signaling pathways, thereby affecting immune regulation.

### 4.6 Study effect and risk of bias

In human observational studies, the LEGEND tool was used to assess the risk of bias. Of the included studies, five were rated as level 4b, one as level 4a, and one as level 3b (**Table 6**). Most of these studies had relatively small sample sizes and did not perform power analyses, indicating potential limitations in statistical validity, but supporting mechanistic research.

**Table 6:** Summary of study designs and LEGEND grades for included human studies.

Author, year	Type of study	Legend grade
Altmäe, 2017 (42)	Cross-sectional study	4b
Allbrand, 2022 (39)	Cross-sectional study	4a
Aye, 2014(41)	Cross-sectional study	4b
Shook,2023 (42)	Cross-sectional study	4b
Méndez-Mancilla, 2018 (43)	Cross-sectional study	4b
Roberts, 2011 (44)	Cross-sectional study	4b
Broadney, 2016 (40)	Prospective cohort study	3b

**Figure 3:**
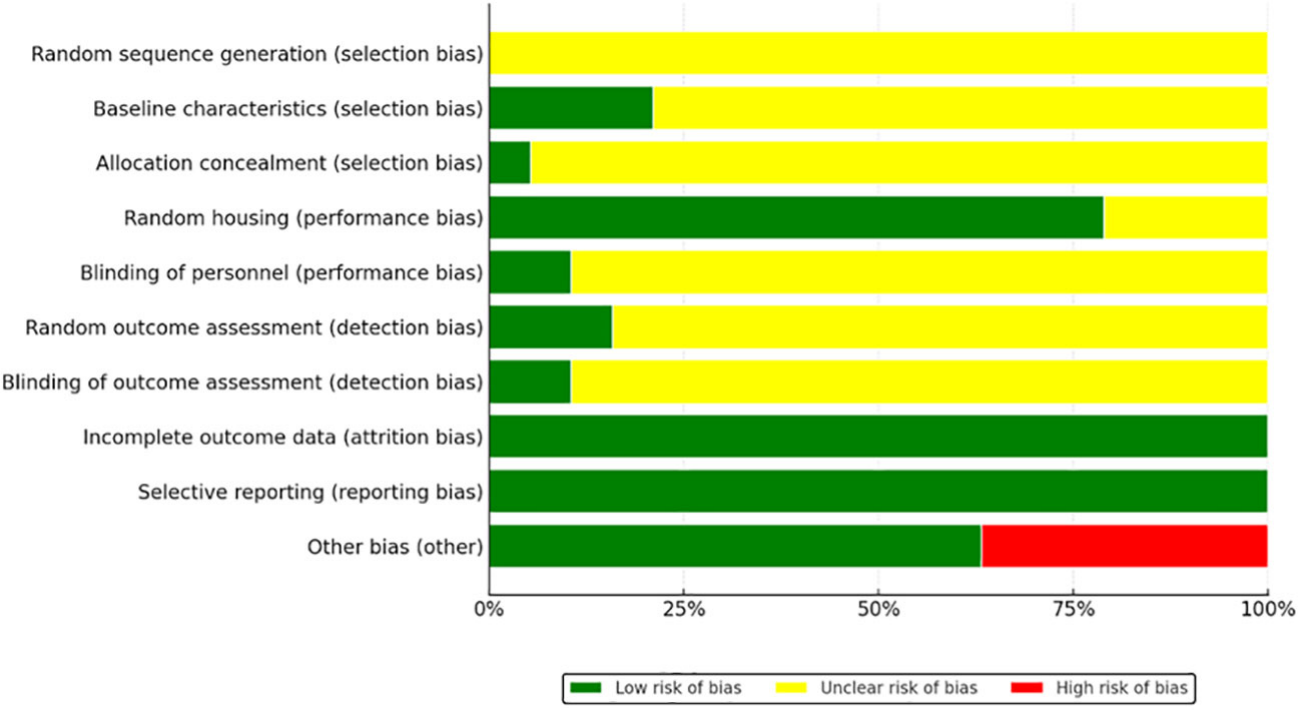
SYRCLE risk of bias across included animal studies.

According to the SYRCLE risk-of-bias assessment, methodological shortcomings were across domains (**Figure 3**). Except for Incomplete outcome data and Selective outcome reporting, most domains exhibited methodological deficiencies. None of the 19 studies adequately described random sequence generation (several merely stated random assignment without detail). In addition, some studies lacked baseline measurements in dams before the intervention, increasing the risk of selection bias. Allocation concealment, blinding of personnel, random outcome assessment, and blinding of outcome assessment were predominantly rated unclear because procedural details were not reported. Several studies included only male offspring, introducing sex bias; others did not report group sample sizes or used small samples without *a priori* sample-size calculation, limiting statistical power and generalizability.

## 5 Discussion

The review found inconsistent associations between MO and offspring birth weight across the included studies. In human studies (n=6), two reported significantly higher birth weight, three reported no significant association, and one did not report a p-value. No human study reported significantly lower birth weight. In animal studies (n=9), one reported significantly higher birth weight, three reported significantly lower birth weight, and five reported no significant association. This inconsistency contrasts with the findings of Koepp et al., who reported that the impact of maternal weight before pregnancy on neonatal outcomes is minimal (76). They further proposed that such effects might become more evident during later stages of development, such as childhood or adolescence; however, this remains a hypothesis and is not supported by the data from the present review. Several factors account for the inconsistent birth weight results across studies. Included studies excluded pregnancies with complications such as gestational diabetes mellitus (GDM) or preeclampsia. GDM is typically associated with higher birth weight, including macrosomia or large-for-gestational-age (LGA), whereas preeclampsia is more commonly linked to small-for-gestational-age (SGA) or fetal growth restriction (FGR) (77, 78). By excluding pregnancy complications, the included populations may represent a metabolically milder obese phenotype with relatively preserved fetal growth. This could attenuate detectability of MO-related birth weight effects, potentially explaining the null or heterogeneous findings in three human studies. There was substantial variability in the countries and regions of origin, with most studies conducted in European populations. Differences in dietary patterns and obesity classification criteria between populations (e.g., different BMI thresholds for obesity in Asian vs. European populations) were not consistently addressed, potentially affecting the comparability of results (79). Additionally, risk of bias analysis indicated that human studies generally had small sample sizes, limiting their representativeness for the general population. Only one human study was rated as high quality (39), whereas the remaining studies were classified as low quality. However, the study by Allbrand et al. included only participants with obesity and lacked a non-obese comparison group, limiting its ability to directly address differences between MO and NMO. Broadney et al. enrolled women across the BMI spectrum (underweight to >35 kg/m²) but did not report differences between obese and normal-weight groups; the extreme underweight-obese contrast further complicates interpretation. Further examination of study characteristics indicated that animal studies showing lower offspring birth weight utilized maternal dietary models with fat-derived energy proportions below 45%. In one animal study (Payolla et al., 58), the duration of high-fat diet exposure prior to mating was not clearly specified. In contrast, animal studies showing higher birth weight more commonly employed high-fat diets with ≥45% energy derived from fat and frequently used the C57BL/6 mouse model. Such inconsistencies in findings may reflect the potential biases of the included studies. In addition, the absence of baseline measurements in most studies may have resulted in unequal starting body weights. All studies included in this review were selected based on the availability of immunology data rather than birth weight as a primary outcome, and the robustness of the birth weight data may thus be lower than that of studies specifically designed for this endpoint.

**Figure 4:**
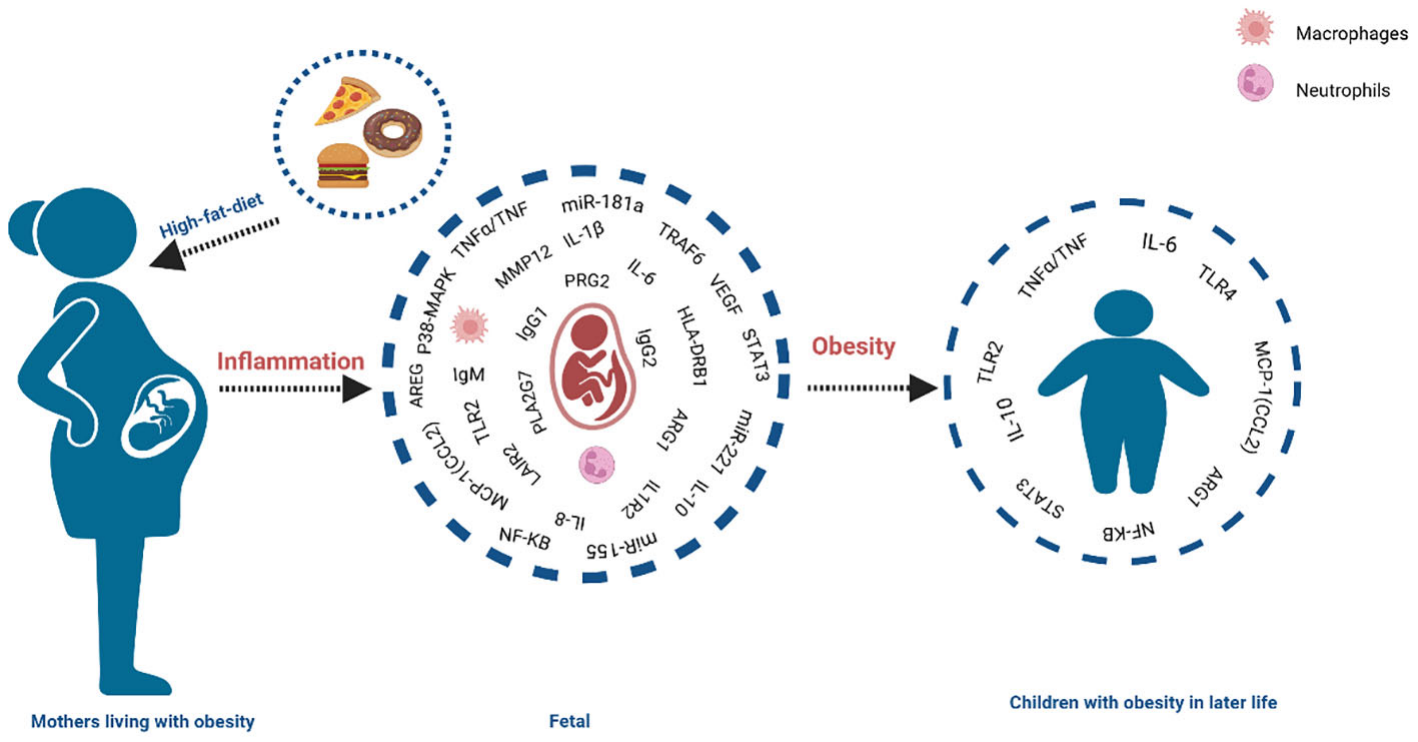
Summary of immune alteration parameters in offspring of mothers affected by obesity. Created in https://BioRender.com.

Building on the observed immune alterations (**Figure 4**), we further identified activated placental inflammatory signaling pathways associated with MO. Included animal studies reported altered expression levels of TLR2, MyD88, TRAF6, NF-κB, MCP-1, TNF-α, IL-6, and IL-10 in placental tissue. These findings suggest a potential interaction between TLR2 and MyD88 that may activate downstream TRAF6 signaling (36, 80). This axis canonically may lead to NF-κB-mediated pro-inflammatory cytokine expression (e.g., TNF-α, IL-1β, MCP-1) and the p38-MAPK-mediated regulation of IL-6 and IL-10 (80) (71, 81). Pro-inflammatory cytokines such as TNF-α, IL-1β, and IL-6 may also activate the TRAF6-TAK1 axis through their respective receptors, triggering the same downstream signaling cascades (80). Notably, human studies have reported altered expression of the p38 MAPK pathway. Beyond the signaling cascade, this review remains unclear which placental immune cell populations drive TLR2-related signaling responses. HBCs are fetal-origin placental macrophages, whereas decidual macrophages are maternal immune cells located within the decidua. These cell populations may exert distinct immunological functions within the placental microenvironment. Shook et al. reported an increase in CD163^+^ HBCs in human placentas from MO, whereas Roberts et al. observed enhanced neutrophil infiltration within the human placental stroma. TLR2 has been detected on the surface of both HBCs and decidual macrophages, suggesting that either population may be responsive to TLR2-mediated stimuli (82, 83). In addition, pro-inflammatory cytokines such as TNF-α, IL-6, and IL-10 are produced by both decidual macrophages and HBCs, suggesting potential cooperative roles in placental inflammation (84). The reviewed evidence suggests that MO may be associated with activation of placental inflammatory pathways involving TLR-related signaling, although direct experimental validation of a unified signaling cascade remains limited. Methodological limitations further complicate interpretation. The animal studies involved multiple species, with considerable variation in sampling time points, sample sources, and detection methods. Moreover, several studies were unclear in key methodological domains, such as random allocation, blinding of personnel, random housing, and outcome assessment. These limitations may partly explain the divergent trends observed for the same immune parameters. Due to the limited number of human observational studies, a comprehensive elucidation of the underlying mechanisms remains challenging.

Based on the above findings, this review further synthesizes a unified mechanistic model: MO may trigger the activation of NF-κB and p38-MAPK signaling pathways within placental macrophages, contributing to fetal inflammation. Upon exposure to endogenous stimuli such as free fatty acids and high glucose, placental macrophages are likely to activate the TLR-mediated MyD88–TRAF6 signaling pathway, subsequently triggering downstream NF-κB and p38 MAPK cascades (85). These pathways work synergistically to induce the expression of multiple pro-inflammatory cytokines. TNF-α, IL-6, IL-10, and MCP-1 are well-established target genes of NF-κB and p38 MAPK signaling in macrophages (86). Sustained NF-κB activation may enhance the pro-inflammatory response and promote the recruitment of neutrophils through chemokines such as MCP-1 and CXCL1. Previous studies have shown that in ovine models, MO may activate NF-κB and JNK pathways in HBCs through TLR4 signaling triggered by circulating free fatty acids (35). Similarly, in murine models exposed to high glucose, activation of the TLR4–MyD88–NF-κB pathway has been observed, further supporting the involvement of inflammatory signaling in placental dysfunction (85).

**Figure 5:**
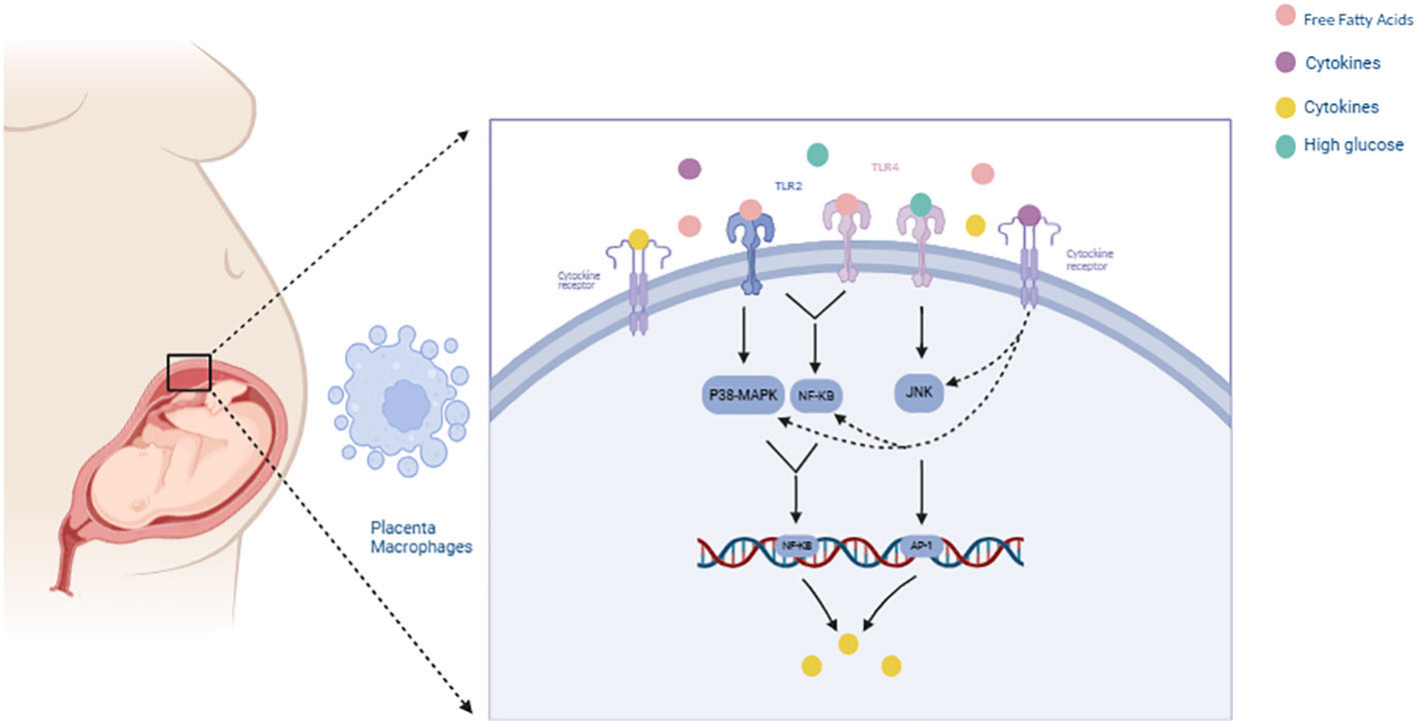
A proposed mechanism, synthesized from the included studies, indicating how MO may contribute to placental inflammation. Created in https://BioRender.com.

This section provides new insights into the potential regulatory mechanisms by which maternal obesity influences placental inflammation. The potential crosstalk and interactions between these signaling pathways are illustrated in **Figure 5**. It is vital to note that this section is based primarily on molecular expression data and lacks functional validation of the proposed mechanism. Therefore, alternative mechanisms cannot be ruled out. Future studies should employ both animal models and human samples to verify the causality of these findings and to further evaluate their impact on fetal development, as well as on possible clinical intervention strategies. Such strategies may include nutritional approaches with antioxidant or anti-inflammatory properties, or broader interventions aimed at improving maternal metabolic and inflammatory status. However, the safety, timing, and efficacy of these approaches require rigorous evaluation in well-designed clinical studies before any translational application can be considered.

## 6 Conclusion and future work

The findings of this systematized review suggest that MO does not necessarily lead to increased neonatal birth weight. However, it may trigger placental inflammation by activating the TLR2, NF-κB, and p38 signaling pathways in placental macrophages, thereby disrupting the local immune environment. Beyond the placenta, MO is associated with broad changes in the developing immune system of the offspring. These include altered expression of cytokines and chemokines, shifts in immune cell populations and antibodies, as well as dysregulation of immune-related genes, microRNAs, and inflammatory signaling molecules. These immune alterations may increase the child’s risk of obesity, allergies, or other immune-related disorders later in life.

Future research should prioritize longitudinal studies that track offspring health outcomes over time, along with mechanistic experiments such as *in vitro* studies or animal models to validate the role of NF-κB, p38 signaling, and specific macrophage subtypes in MO-associated placental inflammation. Current limitations also are addressed in the evidence based to establish a clearer causal link between MO and offspring immune development. As most available studies are observational and heterogeneous in exposure definitions and immune measurements, large-scale longitudinal cohorts are needed to track immune trajectories from preconception to childhood. Mechanistic studies, including *in vitro* experiments and animal models, are warranted to validate the role of NF-κB, p38 signaling, and distinct macrophage subtypes in MO-associated placental inflammation.
